# Knowledge, attitudes and practices of health care providers trained in responding to violence against women: a pre- and post-intervention study

**DOI:** 10.1186/s12889-021-12042-7

**Published:** 2021-11-01

**Authors:** Sanjida Arora, Sangeeta Rege, Padma Bhate-Deosthali, Soe Soe Thwin, Avni Amin, Claudia García-Moreno, Sarah R. Meyer

**Affiliations:** 1 CEHAT – Centre for Enquiry into Health and Allied Themes, Mumbai, India; 2 Department of Sexual and Reproductive Health and Research, World Health Organization, Geneva, Switzerland

**Keywords:** Violence against women, Training, Health care providers, India; tertiary health care; health system

## Abstract

**Background:**

Violence against women is a serious public health concern, and is highly prevalent globally, including in India. Health-care providers [HCPs] can play an important role in addressing and reducing negative consequences of violence against women. We implemented a pre-post intervention study of HCP training in three tertiary care facilities in Maharashtra, India.

**Methods:**

The study used a pre-post intervention design with assessment of HCPs’ (*n* = 201) knowledge, attitudes, perceived preparedness and practice at three time points: before training, after training and at 6 months follow- up.

**Results:**

Total median score of knowledge about common signs and symptoms of violence (8.89 vs, 10.00), attitudes towards acceptability of violence (9.05 vs. 10.00), individual (6.74 vs. 10.00) and system level preparedness (6.11 vs. 8.14) improved from pre to post- training. The generalized estimating equation [GEE] model, adjusted for age, sex, site and department, showed an improvement in knowledge, attitudes and preparedness post- training. The change from pre to 6 months follow- up was not significant for attitude.

**Conclusions:**

This package of interventions, including training of HCPs, improved HCPs’ knowledge, attitudes and practices, yet changes in attitudes and preparedness did not sustain over time. This study indicates feasibility and positive influence of a multi-component intervention to improve HCP readiness to respond to violence against women in a low-resource setting. Future phases of intervention development include adapting this intervention package for primary and secondary health facilities in this context, and future research should assess these interventions using a rigorous experimental design. Finally, these results can be used to advocate for multi-layered, systems-based approaches to strengthening health response to violence against women.

**Supplementary Information:**

The online version contains supplementary material available at 10.1186/s12889-021-12042-7.

## Background

Violence against women is a pervasive and highly prevalent health and social problem, with estimates showing that almost one-in-three women globally have experienced physical and/or sexual violence by an intimate partner or non-partner sexual violence in their lifetime [1]. It is a public health problem with significant implications for women’s mental, physical and social well-being [2]. There is well-documented evidence which suggests strong associations between violence against women and health consequences including sexually transmitted infections, HIV, unwanted pregnancy, miscarriage, injuries, depression, suicidal ideation, substance abuse and chronic pain [3–7].

Intimate partner violence [IPV] is the most prevalent form of violence against women. In India, the fourth round (2015–16) of National Family Health Survey (NFHS) found that 31% of ever married women have been subjected to physical or sexual violence by their husband in their lifetime. Among women who have experienced partner violence, only 14% have sought any form of formal or informal support following such violence [8]. This is despite widespread evidence that suggests that both formal and informal support systems can play a crucial role in mitigating consequences of violence against women [9, 10].

Health systems have an important role to play in a coordinated multi-sectoral response to violence against women [11]. Women facing violence have frequent contacts with health systems. Even if women do not disclose violence to healthcare providers [HCPs], HCPs are in an ideal position to identify and respond to women facing violence [12–14]. However, there are significant barriers that prevent HCPs from identifying abuse and providing appropriate care and support to women affected by violence [15, 16]. Studies have suggested HCPs’ lack of awareness, skills, prejudicial attitudes towards and stereotypes of violence against women as major factors responsible for preventing abused women to access quality healthcare [17, 18]. For example, a study in Kenya found that providers’ understandings of violence against women were based primarily on their experiences of addressing non-partner sexual violence. This had implications for their (lack of) willingness to identify and provide appropriate response for women experiencing IPV [19]. In the Indian context, these barriers are evident from the fact that only 1% of married women who have ever faced violence from a husband sought any support from an HCP, although quarter of women reported injuries as a consequences of violence, thus indicating that even in case of injuries, survivors may not disclose violence [8].

In-service training is one of the widely suggested ways to address barriers faced by HCPs in responding to violence against women [20, 21]. In order to provide the basis of a comprehensive health system response to violence against women, these training programs should influence providers’ beliefs and increase HCPs’ knowledge and skills to respond to women facing violence, while ensuring their safety and protecting their confidentiality [11]. There is some evidence of effectiveness of training interventions using interactive techniques [22]. A pre and post-intervention study of a training intervention for public health midwives on response to IPV in Sri Lanka found that role plays, field handbooks and cultural sensitivity training were important components of the intervention [23]. The training intervention significantly improved midwives’ skills in identifying women affected by violence, improved midwives’ knowledge of violence against women and decreased perceived barriers to supporting women affected by violence.

In low and middle-income countries [LMICs], there are numerous challenges in conducting in-service training of HCPs to improve healthcare response to violence against women [24, 25]. These include gender blind medical education, HCPs’ lack of time and heavy patient- load, and a high turnover of HCPs in health facilities. Furthermore, there are several system level constraints, such as inadequate numbers of available health workforce, limited infrastructure and lack of support services for referrals, which need to be addressed in order to improve quality of the health system response [26]. Studies suggest that in order to strengthen health system response to violence against women, training of healthcare providers alone is not sufficient [27–29]. For example, in a study of a training intervention for general practitioners and residents in general practice in Greece, the intervention resulted in increase of knowledge and self-preparedness but it did not translate into significant changes in clinical practice, indicating the need for systems-level changes for sustainable improvements in clinical practice to be effected by training of HCPs [27]. Systems-level support include establishment of standard operating procedures, referral linkages, building leadership support, supportive supervision and availability of adequate infrastructure for ensuring privacy and confidentiality.

There is little evidence on characteristics, methodologies and effectiveness of training interventions adequate in meeting needs of healthcare providers in LMICs [30]. There are also gaps in understanding of how to implement systems readiness activities (e.g. infrastructure, management support) to further sustain changes health care providers’ abilities to retain knowledge and skills and improve their clinical practice in responding to violence against women. In response to this urgent public health problem, the World Health Organization [WHO] published clinical and policy guidelines, *Responding to intimate partner violence and sexual violence against women* in 2013 [henceforth, the Guidelines], to strengthen the health care provider capacity and health system readiness to respond to violence against women [31]. The Guidelines provide evidence-based recommendations to equip HCPs on *what* to do to respond to intimate partner violence and sexual violence against women. WHO has published two tools to translate the guidelines into practical *“how to”* instructions and job aids. One is a clinical handbook for health care providers, *Health care for women subjected to intimate partner violence or sexual violence (2014)* [32]*.* The second is a manual for health managers, *Strengthening health systems for women subjected to intimate partner violence or sexual violence (2017)* [33]. There remain significant gaps in knowledge of how to implement and facilitate the uptake of these tools in order to effectively improve the performance of HCPs and health system/service readiness.

To address these gaps, CEHAT (Centre For Enquiry Into health and Allied Themes), a Mumbai-based research organisation, collaborated with WHO to explore approaches to implement the Guidelines and WHO tools in three tertiary hospitals of Maharashtra, India, through a multi-component implementation research project.

We conducted a mixed-methods study piloting the implementation of the Guidelines and WHO tools, in order to improve understanding of local contextual factors influencing implementation, including/particularly training, intervention outcomes and support future scale-up. The specific objectives of the over-arching study were:

1. To validate approaches to roll out the training and service delivery improvement activities based on the Guidelines and associated tools by:
  - assessing needs and priorities of health care providers and managers in responding to violence against women;
  - adapting, implementing the training and assessing improvements in provider knowledge, attitudes, perceived preparedness and practice
  - assessing the relevance of the training approaches in meeting the needs of health care providers and identifying barriers and facilitators for health care providers to deliver care to women subjected to violence
2. To understand the perceptions of quality of care of women subjected to violence who have received care from trained health care providers.
3. To develop, validate and refine instruments for measuring health care providers’ performance and health system/service readiness instrument

This manuscript reports on findings from Objective 1b specifically, we seek to assess: i) if the training intervention improved HCPs’ knowledge, attitudes and practices related to responding to violence against women, ii) if those improvements were maintained between post-training and 6-month follow-up, and iii) if age, sex, department and site of the HCPs was associated with changes in knowledge, attitudes and practices.

## Methods

The study used a pre and post-intervention design with assessment of HCPs’ knowledge, attitudes, perceived preparedness and practice at three time points: before training, immediately after training and 6 months follow- up. This study is the pilot stage of a multi-phase research project, and various aims (listed above), were a first step prior to our plans to expandi the intervention and conduct an impact evaluation using an experimental design. In this pilot phase, we sought to first see if the intervention could be adapted and was feasible to implement in this context, meaning that HCPs attended the trainings and demonstrated measureable changes in aspects of clinical practice addressed in the training. This study design was selected to ensure the most rigorous design while taking into account the range of challenges present in implementing training and assessing training outcomes in this context.

### Setting

This study was conducted in two districts in the state of Maharashtra, India between July 2018 and April 2019. The study was carried out at three tertiary medical teaching hospitals: Aurangabad Government Medical College, Aurangabad and Miraj Government Medical College and Sangli District Hospital. These facilities were identified based on their participation in a prior collaborative project with CEHAT on integrating gender within medical education [34], implemented in these and five other hospitals in the state of Maharashtra. The selected hospitals were attached to the medical colleges that had performed best in terms of integrating modules on gender in pre- service curriculum. Further, they were selected as there were at least two gender sensitive medical educators in these facilities who could lead the implementation of the intervention. These hospitals were identified as being the most suitable for this study because integration of gender within medical education can be utilized as a foundation upon which to further build capacity related to violence against women. However, there is still great diversity within and across the hospitals in both districts in terms of capacity and readiness to respond to VAW.

Aurangabad district has a population of about 3.7 million and the Government medical college is biggest tertiary care hospital for central Maharashtra [35]. It has more than 1000 beds and serves as a referral point for both urban and rural population. The average flow of patients on outpatient basis is about 27,000/month and about 5600/month as in- patient visits [36]. Sangli district has a population of about 2.8 million with 75% of the population being rural [37]. Miraj medical college has 320 beds while a district hospital managed by college has a total of 380 beds. On average, 52,000 patients visit Miraj medical college and Sangli district hospital every month on an outpatient basis [38].

### Participants

The training participants were selected based on the following criteria: i) HCPs providing services to patients in any of the following three departments: Obstetrics & Gynecology, General Medicine and Casualty/ Emergency. These three departments were selected as more women access clinical care in these departments and HCPs who were less likely to be transferred from study sites for the duration of the study.

Given that this study was conducted as part of a formative phase of research, the sample size in this study was not based on power calculations. Rather, it was determined based on the feasibility of including the largest number of healthcare providers in the training given their availability and interest in developing skills to respond to violence against women. We estimated that a minimum of 30% of healthcare providers could take part in the training and be retained and feasibly followed up over the three time-points. This gave us an approximate sample size of 170 HCPs which was further increased to 220 to account for an expected 20% of attrition of providers at the 6 month follow-up assessment.

### Intervention

The training approach employed the following steps. A cascade training approach was employed, wherein a selected group of senior administrators of the selected departments were trained as master trainers. These master trainers were trained over a period of five days by experts such as other healthcare providers, as well as lawyers, academicians and women’s rights activists experienced in training healthcare providers on violence against women. The master trainers then then trained other HCPs in their own facilities, both their peers as well as junior providers. A total of eight two-day and eight half-day peer led refresher trainings were conducted by trainers at their respective health facilities. A maximum of 30 participants were included in each training and a mix of doctors, nurses and social workers were trained together. The trainings were planned in advance so that a arrangements could be made to cover routine clinical service provision. The rationale for including different cadres together in training sessions was to minimize inter-professional cadre hierarchies that exist between doctors, nurses and social workers, create an across health professional cadre team approach, and allow for triaging survivors needing care in accordance with role/function of each cadre and their time availabilities to carry out certain tasks related to responding to violence. The training was built on a draft curriculum manual developed by WHO, based on the Guidelines, and CEHAT’s curriculum [39], which reflects its decade-long work with the Indian public health sector on violence against women. The training content was translated in Marathi and was also made available as a manual to trainers. Table 1 shows topics included in the modules implemented in the training.

**Table 1:** Modules and concepts addressed in the training

Knowledge, Attitudes and Preparedness	Themes covered in training	Topics included under themes
Knowledge and attitude oriented topics	Awareness about violence against women	Prevalence, forms, and health consequences of violence, VAW as a public health issue
	Concepts related to violence against women	Difference between sex and gender, gender norms, patriarchy, intersectionality, myths about VAW
	Role of HCPs in responding to VAW	Signs and symptoms indicating violence, legal mandate of healthcare providers in India, Identification by healthcare providers-ways to ask
Skills in clinical care	Establishing ideal response of HCPs	Provision of first line support – Listen, Inquiry, Validate, Enhanced Safety and Support (LIVES) Documentation of cases

Participatory methods including role plays, clinical case studies, vignettes, and games were used by trainers to deliver the training. At one site, the trainers invited protection officer to jointly conduct the session on the legal mandate of healthcare providers. At the end of the training, a pocket-sized reminder card describing steps for providing first line support to women was given to each participant.

Additionally, several system level changes were implemented to enable HCPs to apply the skills they learned during the training and sustain changes in clinical practice. These system changes included: i) establishment of standard operating procedures for establishing privacy, confidentiality, clinical care and documentation of cases; ii) establishment of referral linkages by organizing a meeting between healthcare administrators and organizations providing support services; iii) creating a referral directory for healthcare providers; iv) Introducing a one page documentation register as part of the health management information systems to enable HCPs to document case of violence; v) creation of job-aids for providing care, documentation and maintaining privacy and confidentiality; and vi) discussion of care and support for domestic violence survivors (‘case management’) in clinical meetings with HCPS to facilitate supervision, mentoring and peer-to-peer learning for other HCPs in the facility.

### Study instrument

A self- administered, paper-based structured questionnaire was used to assess baseline, and changes in HCPs’ knowledge of, attitudes towards, perceived preparedness and clinical practices regarding violence against women, immediately after the training and 6 months after the training. The survey instrument was developed by incorporating relevant items from the Physician Readiness to Manage Intimate Partner Violence Survey tool [PREMIS], the Domestic Violence Healthcare Provider Survey Scales [DVHPSS] and the Demographic and Health Surveys [DHS] questions on attitudes towards violence against women. The PREMIS and DVHPSS are existing instruments designed to measure HCPs’ preparedness to respond to survivors of intimate partner violence [40]. The PREMIS has been validated in the United States of America and the DVHPSS has been validated in Uganda and Nigeria [41]. Items pertaining to Indian legal frameworks, such as an item on provisions of Protection of Women from Domestic Violence Act, 2006, were added to adapt to the local context.

The instrument was translated into Marathi and piloted with a sample of 20 doctors and nurses working in a tertiary hospital in Mumbai, Maharashtra. The results of the pilot test indicated that some changes were needed, including improving clarity of some items, and further adapting other items to the local context. For example, the term “intimate partner violence” was replaced with the term “domestic violence” throughout the tool, as legal and policy frameworks in India refer to domestic violence to indicate domestic relationships that go beyond an intimate partner and capture violence perpetrated by in-laws and other family members. The item “It is important not to share or discuss the woman’s information with anyone unless she authorizes it” was changed to “It is important not to share or discuss the woman’s information with anyone unless she consents to it,” as the word “authorize” was not clear to providers.

We calculated Cronbach’s alpha for various domains based on baseline data, and considering these results, we dropped some questions focused on gender norms and perceptions of the role of healthcare providers for the purpose of this analysis due to very low values of Cronbach’s alpha. The following constructs were analysed for this manuscript:

i. **Knowledge:** Knowledge in this analysis was measured using 15 items with response as yes, no or don’t know. Each correct answer was given a score of 1, while incorrect and don’t know response were given a score of 0. Knowledge was assessed using two sub-domains: knowledge of clinical signs and symptoms indicating violence, and knowledge of appropriate ways to ask about violence. Examples of knowledge related items are: “Which of the following are warning signs that a woman may have been subjected to sexual violence or domestic violence?” Repeated unwanted pregnancy? - Yes/No/ Don’t know and Alcohol or drug abuse? - Yes/No/ Don’t know, and “Which of the following are the most appropriate ways to ask about domestic violence?” – “Are you a victim of domestic violence?” - Yes/No/ Don’t know and “Has your partner ever hurt or hit you?” - Yes/No/ Don’t know
ii. **Attitudes:** Attitudes were measured by a total of 13 items. The attitudes of providers were assessed by using the Demographic and Health Survey Domestic Violence module items on acceptability of violence against women (7 items) and items assessing attitudes towards asking women about violence (6 items) (the Professional Role Resistance/Fear of offending the Patients sub-scale from the DVHPSS). The acceptability of violence against women was measured with responses: Never acceptable/Sometimes acceptable/Acceptable and the score ranged from 3 to 1, where Never acceptable was given score 3. Attitudes towards role of HCPs was measured using 5-point Likert scale. Examples of items assessing attitudes towards role of HCPs asking about violence are: “If I ask non-abused patients about domestic violence, they will get very angry” and “I am afraid of offending the patient if I ask about domestic violence”
iii. **Perceived Preparedness:** Perceived preparedness of HCPs was considered at individual and system level. Items related to system level preparedness like support from colleagues, was captured using responses yes, no or don’t know. Individual preparedness was measured using 5-point Likert scale: Not at all prepared, slightly prepared, somewhat prepared, sufficiently prepared and quite well prepared.
iv. **Practices:** Practice was assessed using items focused on identification of cases in last 3 months and services provided by healthcare providers to women.

The tool administered immediately after the training was the same as the baseline instrument except that it did not include the question on practice in last 3 months. Additional items were added to 6 months follow-up tool to capture perceived need for additional training, facilitators and barriers faced by healthcare providers in responding to women facing violence. Table 2 displays each construct, domain, sources for the items, and the Cronbach’s alpha. The full list of items in each domain is included in Appendix 1.

**Table 2:** **Construct, domains, sources and Cronbach’s alpha of measures**

Construct	Domain	Source	Cronbach’s alpha
Knowledge (15 items total)	Clinical knowledge (9 items)	Adaptation of items from PREMIS	0.70
	Ways to ask about violence (6 items)	Adaptation of items from PREMIS	0.60
Attitudes (13 items total)	Acceptability of violence (7 items)	DHS Domestic Violence Module	0.84
	Attitudes towards asking about violence (6 items)	Professional Role Resistance/Fear of offending the Patients sub-scale, DVHPSS	0.71
Preparedness (15 items)	Individual preparedness (9 items)	Adaptation of items from DVHPSS and PREMIS	0.93
	System-level preparedness (6 items)	Adaptation of items from DVHPSS	0.72

### Data collection

The survey instrument was administered at three points of time: before starting training (i.e. pre-training), immediately after the training (i.e. post-training) and six months after the training (i.e. 6-month follow-up). To increase the participation of providers at post- 6 month assessment, we organized a half day refresher training and the tool was administered before commencing the refresher training. The average time taken by providers to complete the tool was 35 to 40 min. A unique ID number specific to each respondent was used to match each of the three assessments. Following paper-based self-completion of the surveys, CEHAT research team members entered the data into OpenClinica, an online data entry system. We checked accuracy of entered data by comparing paper surveys to entered data randomly.

### Data analysis

We used SPSS Version 20.0 for statistical analyses [42]. We conducted descriptive data analysis to examine and summarize socio- demographic details of participants: age, number of years of clinical experience, department and role within the health facility, i.e. doctor, nurse, social worker. As number of items in each domain were varied and the range of responses was different, we rescaled domains. The rescaling of domains was done so as to have same lower and upper limits (0–10). This was done by computing the score of each of the domains out of 10 i.e. by dividing the original score with the original range and then multiplying by 10. Since there were two domains under each construct, the score of domains were added to calculate scores of constructs. Thus, the scores of knowledge, attitudes and perceived preparedness ranges from 0 to 20. The scores of each domain under each construct were summed (for example, adding the clinical knowledge score and the ways of asking about violence score for a total knowledge score), thus each domain (knowledge, attitudes and practices) ranges from 0 to 20.

The distribution of scores of knowledge, attitudes and preparedness was assessed graphically by plotting histograms, and the Shapiro-Wilk test indicated that all outcome measures were significantly different from the normal distribution. The pair-wise comparison can lead to inflated Type I error, therefore the overall effect of training was assessed using multivariable Generalized Linear Models using Generalized estimating equation (GEE) that were adjusted for age, sex, site and department. The GEE model was also used to present the effect of training on each of the domain. We used an exchangeable correlation matrix with a robust estimator assuming homogeneous correlation between repeated measurements of scores. As dependent variables wereare not normally distributed, gamma log link with type III analysis was used. The main effects model with a three-level indicator for time (pre-, post-, and 6 month follow-up) as independent variable was fitted to estimate change in dependent variable (scores) at post- and at 6 month follow-up. .

All models were adjusted for age of the provider, training site, department of the provider as there is evidence in literature on role of age and sex of the healthcare provider in determining the impact of training (12). As trainers were site specific, site was included in the model to assess any difference in outcomes of training. The three departments differ from each other in terms of patient load and health symptoms with which female patients present. Thus, department was one of the variables included in GEE model.

For analysis purposes, data of HCPs from Miraj and Sangli were pooled because the two hospitals are managed by the same administration and the master trainers who are senior health administrators and health care providers rotate between the two hospitals. The training of HCPs from Miraj and Sangli hospitals was done together and by master trainers who worked and oversaw HCPs in both facilities, therefore in all analyses, outcomes from these two facilities were combined.

### Ethical considerations

The project was reviewed and approved by the Institutional Ethics Committee of CEHAT. The project was also approved by the Research Project Review Panel [RP2], an independent technical review panel of the HRP (the UNDP/UNFPA/UNICEF/WHO/World Bank Special Programme of Research, Development and Research Training in Human Reproduction) at the WHO, and the World Health Organization’s Ethics Review Committee [ERC], which reviews all human subjects research conducted or supported by WHO. Permission to conduct the study was also obtained from Directorate of Medical Education and Research (DMER), Maharashtra, which is the governing body for tertiary teaching hospitals in Maharashtra. Informed consent was obtained from all participants. The informed consent was translated into the Marathi and informed participants about the measures implemented to ensure confidentiality. The unique ID for matching three levels of questionnaire was stored separately from the registration lists of trainings. All methods were carried out in accordance with relevant guidelines and regulations.

## Results

### Characteristics of the study population

Table 3 shows characteristics of study population. The assessment at before training, after training and 6 months following training was completed by 201 of 220 (91.4%) HCPs. 19 HCPs (8.6) were lost to follow-up at 6 months. There was no difference in the socio-demographic characteristics of those lost to follow-up compared to the sample retained at follow-up. Transfer of HCPs from one health facility to another was the most common reason for loss to follow- up. Out of 201 providers, 90 providers were from Aurangabad hospital while 111 were from Miraj-Sangli hospitals. About 54% of providers were nurses or nursing assistants, 41% were doctors and the remaining were social workers. 70% of HCPs were females while remaining were males. About 52% of HCPs were 25 to 34 years of age. The majority of providers (41.3%) were from Obstetrics and Gynecology department, followed by Medicine (36%). The mean number of years of clinical experience of providers was 11.9 years (SD = 9.7) with a range of less than a year to 32 years. There was no significant hospital- wise difference found in socio-demographics of HCPs apart from department of participants, which can be attributed to the small number of participants in the “other departments” category in both sites. The on-site trainings covered all doctors and nurses from the three departments included in this study, therefore the demographic characteristics of participants are representative of all HCPs working in the three departments.

**Table 3:** **Characteristics of the study population**

	Aurangabad N (%)	Miraj- Sangli N (%)	Full sample N (%)	p- value
**Sex**				
Male	27 (30.0%)	33 (29.7%)	60 (29.9%)	0.967
Female	63 (70.0%)	78 (70.3%)	141 (70.1%)	
**Name of department**				
Gynecology	35 (38.9%)	48 (43.2%)	83 (41.3%)	0.034
Medicine	40 (44.4%)	33 (29.7%)	73 (36.3%)	
Casualty	8 (8.9%)	24 (21.6%)	32 (15.9%)	
Other*	7 (7.8%)	6 (5.4%)	13 (6.5%)	
**Job within facility**				
Medical doctor	32 (35.6%)	51 (45.9%)	83 (41.3%)	0.101
Nurse and Nursing Assistant	53 (58.9%)	56 (50.4%)	109 (54.3%)	
Others**	5 (5.6%)	4 (3.6%)	9 (4.4%)	
**Age**				
Less than 25 years old	9 (10.0%)	8 (7.2%)	17 (8.4%)	0.848
25–34 years old	46 (51.1%)	58 (52.3%)	104 (51.7%)	
35–44 years old	19 (21.1%)	23 (20.7%)	42 (20.8%)	
45 years or older	16 (17.8%)	22 (19.8%)	38 (18.9%)	
**Total N (% of full sample)**	**90 (44.8%)**	**111 (55.2%)**	**201 (100%)**	

* A total of *n* = 13 participants were working in other departments at the time of the training (surgery and psychiatry), but were included in the training as they were nurses who rotated into the relevant departments.**Others include social workers and clinical department helpers

### Change in knowledge of providers

Table 4 shows change in median scores of two domains of knowledge - clinical knowledge and appropriate ways to ask about violence. The table also includes GEE estimates for change in clinical knowledge and ways to ask about violence with pre- training as reference. The median score of clinical knowledge of VAW increased from pre to post training (8.89 vs. 10.00) There was no change in median score between post-training and 6-month follow-up. The GEE model adjusted for age, sex, department and site indicated a significant change from pre to post- training (*p* < .001) and pre to 6-month follow-up (p < .001) for both clinical knowledge and ways to ask about violence.

**Table 4:** Change in Knowledge from pre to post and 6-month follow-up

Variable	Pre- training Median (IQR)	Post- training Median (IQR)	6 months follow- up Median (IQR)	B Coefficient (Adjusted) (95% Wald confidence interval)	B Coefficient (Adjusted) Follow- up (95% Wald confidence interval)
Clinical Knowledge	8.89 (3)	10.00 (1.67)	10.00 (1.43)	0.10 (0.07–0.13)*	0.09 (0.05–0.12)*
Ways to ask about violence	8.33 (3)	8.00 (2)	8.33 (3.33)	0.10 (0.03–0.15)*	0.09 (0.04–0.15)*

*GEE estimates are significant at *p* < 0.01

### Change in attitudes of providers

Table 5 shows change in median scores of providers’ attitudes towards acceptability of violence against women and HCPs’ attitudes towards asking women about violence. The median score on attitudes towards acceptability of violence increased (i.e. views that violence was acceptable decreased) from pre to post and this improvement was sustained at 6-month follow-up (9.95 vs 10.00). The adjusted GEE estimates revealed a significant change in attitude towards less acceptability of intimate partner violence from pre to post (*p* < .001) and 6-month follow-up (*p* = .002).

**Table 5:** Change in attitudes about intimate partner violence from pre to post and 6-month follow-up

Variable	Pre- training Median (IQR)	Post- training Median (IQR)	6-month follow-up Median (IQR)	B Coefficient (Adjusted) (95% Wald confidence interval)	B Coefficient (Adjusted) (95% Wald confidence interval)
Acceptability of partner violence	9.05 (4.00)	10.00 (1.43)	10.00 (1.76)	0.10 (0.08–0.14)*	0.06 (0.02–0.10)*
Attitudes towards asking women about violence	5.45 (2)	5.56 (2.78)	5.78 (2.63)	0.03 (−0.02–0.08)	−0.01 (− 0.06–0.05)

*GEE estimates are significant at *p* < 0.01

### Change in HCPs’ perceptions of preparedness

Table 6 shows change in perceived preparedness of HCPs in terms of individual and systems level support. The median scores of both individual level preparedness (6.74 vs. 10.00) and system level support (6.11 vs. 8.14) improved considerably between pre and post-training. However, a decline in median scores was observed from post- training to 6- month follow-up for both individual (10.00 vs. 8.33) and system level preparedness (8.14 vs. 7.04). After adjusting for age, sex, site and department the GEE estimates shows significant increase in individual and system preparedness from pre to post and 6- months follow-up.

**Table 6:** Change in perceived preparedness from pre to post and 6-month follow-up

Variable	Pre- training Median (IQR)	Post- training Median (IQR)	6 -month follow-up Median (IQR)	B Coefficient (Adjusted) (95% Wald confidence interval”	B Coefficient (Adjusted) (95% Wald confidence interval)
Individual level preparedness	6.67 (4)	10.00 (2.96)	8.33 (1.67)	0.29 (0.23–0.35)*	0.25 (0.19–0.31)*
System level support	6.11 (4)	8.14 (2.96)	7.04 (3.33)	027 (0.22–0.34)*	0.18 (0.12–0.24)*

*GEE estimates are significant at *p* < 0.01

### Change in practice of providers to identify and provide services to women facing violence

Table 7 shows identification of survivors in last 3 months by HCPs and provision of different kinds of support services before training and 6 months later. Six months post- training, 72.1% of providers had identified at least one survivor in the last 3 months as compared to 48.8% before training (*p* < .001). This increase in identification was not found to be significant for providers from Aurangabad hospital (24.4% vs. 25.9%, *p* = .385, *n* = 90). A highly significant increase in identification was found for female providers (35.5% vs. 52.7%, *p* < .001, *n* = 141) and providers from Miraj- Sangli (24.4 vs. 46.3%, p < .001, *n* = 101). At 6-months follow-up, a two- fold increase in the provision of support services like provision of basic information to woman about violence (32.3% vs. 68.7%), discussing options with women (32.8 vs. 70.6%), helping woman to develop a safety plan for (24.9% vs. 51.2%) and referral to support services (25.4% vs. 58.7%) was found. All of these differences were significant at the *p* < .001 level.

**Table 7:** Change in practice

Variable	Pre- training	6-months follow-up	***p***-value
**Actual practice**	**n (%)**	**n (%)**	
**In last 3 months have identified a woman facing violence**	98 (48.8%)	145 (72.1%)	< 0.001
Male (*n* = 60)	27 (13.4%)	39 (19.4%)	0.026
Female (n = 141)	71 (35.3%)	106 (52.7%)	< 0.001
Aurangabad (n = 90)	49 (24.4%)	52 (25.9%)	0.325
Miraj- Sangli (*n* = 111)	49 (24.4%)	93 (46.3%)	< 0.001
**Services provided**			
Provided basic information about domestic violence to the woman	62 (32.3%)	138 (68.7%)	0.015
Offered validating and supportive statements	83 (41.3%)	143 (71.1%)	0.003
Talked to the woman about her needs	72 (35.8%)	138 (68.7%)	0.015
Discussed the options she may have	66 (32.8%)	142 (70.6%)	0.019
Documented domestic violence history and physical examination findings in patient’s chart	52 (25.9%)	111 (55.2%)	0.008
Assessed the immediate level of danger for the woman	60 (29.9%)	116 (57.7%)	0.008
Helped the woman to create a plan to increase her and her children’s safety	50 (24.9%)	103 (51.2)	0.002
Provided education or resource materials about domestic violence to the woman **(**pamphlets, brochures, etc.)	25 (12.4%)	62 (30.8%)	0.002
Referred the woman to support services available within the community (psychological, legal, shelter, etc.)	51 (25.4%)	118 (58.7%)	0.007

Further, we also analysed the improvement in the practice of those HCPs who reported identifying cases of violence before training (n = 81, Table 8). Amongst these providers, a highly significant (*p* < .001) improvement in provision of support services like providing basic information about domestic violence (64.2% vs. 95.1%), offering supportive statements (81.5% vs. 100%), documentation of cases (51.9% vs. 79%) and making external referrals (53.1% vs. 81.5%) was found.

**Table 8:** Improvement in practice of providers who reported identifying women at baseline and 6 month follow-up (*n* = 81)

Variable	Pre- training	6-month follow-up	***p***-value
**Services provided**	**n (%)**	**n (%)**	
Provided basic information about domestic violence to the woman	52 (64.2%)	77 (95.1%)	< 0.001
Offered validating and supportive statements	66 (81.5%)	81 (100%)	< 0.001
Talked to the woman about her needs	60 (74.1%)	77 (95.1%)	0.001
Discussed the options she may have	53 (65.4%)	80 (98.9%)	< 0.001
Documented domestic violence history and physical examination findings in patient’s chart	42 (51.9%)	64 (79%)	< 0.001
Assessed the immediate level of danger for the woman	47 (58%)	68 (84%)	0.001
Helped the woman to create a plan to increase her and her children’s safety	40 (49.4%)	56 (69.1%)	0.008
Provided education or resource materials about domestic violence to the woman (pamphlets, brochures, etc.)	19 (23.5%)	31 (38.3%)	0.038
Refer the woman to support services available within the community (psychological, legal, shelter, etc.)	43 (53.1%)	66 (81.5%)	< 0.001

### Results of generalised estimating equation

Table 9 shows the results of the GEE model on change in overall scores from pre to post and from pre to 6 months for knowledge, attitudes, and practice. A multivariable generalised GEE model was fitted with knowledge, attitudes and practice scores as dependent variables and time, department, sex, age, centre and site as independent variables. The GEE model indicated that change in scores from pre training to post training was significant for knowledge, attitudes, and perceived preparedness. The change in scores from pre to 6 months follow- up was not found to be significant for attitude. Our findings indicate that the training intervention improved knowledge, attitudes and practices of HCPs, with variation in changes in these domains at different time points. In the unadjusted model, the change in knowledge, attitudes and perceived preparedness were found to be same as that in adjusted model. This indicates that age, sex, site and department have no effect on the amount of change in knowledge, attitudes and perceived preparedness of providers over time.

**Table 9:** GEE model adjusted for sex, site, age, department

		Adjusted Change in Scores	
	Knowledge Estimate (95% Wald confidence interval	Attitudes Estimate (95% Wald confidence interval	Preparedness Estimate (95% Wald confidence interval
**Intercept**	2.83 (2.76–2.90)*	2.83 (2.74–2.91)*	2.40 (2.27–2.52)*
6-month follow-up	0.09 (0.05–0.13)*	0.04 (0.00–0.11)	0.25 (0.20–0.30)*
Post- training	0.09 (0.05–0.13)*	0.08 (0.05–0.11)*	0.32 (0.26–0.37)*
Pre- training	Reference	Reference	Reference

*GEE estimates are significant at *p* < .0.01

## Discussion

This pilot study reports on the influence of a training intervention on knowledge, attitudes, and skills of HCPs to ask about violence, provide first-line support and enable provision of social and legal support through referrals. The intervention in this study includes both training and system level changes to create a supportive ecosystem for HCPs to respond to VAW. Various organisational changes such as establishing protocols, mentoring by senior clinicians, and establishing referral linkages were introduced to enable trained HCPs to respond to violence against women in their clinical practice. Some of these system-level changes were also integrated into training, for example delivery of training by clinicans with managerial responsibilities ensured mentorship. The presence of stakeholders involved in providing external support services helped in building capacities of HCPs for making external referrals.

This study fills an important gap in literature as there are few interventions for improving HCP response to violence against women but there are few with an evaluation component [43]. Further, the majority of training interventions that have been assessed have been implemented in North America, with a very limited evidence-base from LMICs particularly in Asia [44]. For example, a recent systematic review of trials of HCP training (comparing interventions to a wait-list or placebo group) to improve IPV response found that of the 19 included studies, three quarters of all studies were conducted in the USA, and no studies were conducted in Asia [45]. The common outcomes measured by the studies included knowledge, beliefs, self- confidence, skills of healthcare providers and patient related outcomes like perceptions of women about the services provided by HCPs [44].

The findings of the present study have indicated a significant increase in overall knowledge, supportive attitudes towards survivors and individual HCP preparedness following training, however, change in attitudes between pre-training and 6 month follow-up was not significant. This gain of knowledge and skills were also reflected in the significant increase in proportion of HCPs identifying and responding to cases of violence, as well as other supportive practices, such as offering vaidating and supportive statements and talking to women about their needs. This is an important finding because other evaluation studies have reported mixed findings for change in identification and response to survivors by HCPs [46]. As the intervention included both training and organisational changes, this study found the largest magnitude of change in perceived preparedness when we compared pre- training, post- training and 6 months follow- up scores. Further, our study found that the change was retained for knowledge whereas for attitudes and perceived preparedness, the change was not sustained over time. This finding indicates that bringing and sustaining change in attitudes and beliefs of providers requires ongoing reinforcement and further training. Also, our findings indicate that changes in different aspects of attitudes vary. The attitudes of HCP towards acceptability of violence changed between pre-training and post-training, and pre-training and 6 month follow-up. However, attitudes towards the role of HCPs in asking about violence did not change between pre-training and post-training, and pre-training and 6 month follow-up. These findings are consistent with the literature [30, 46], and this finding also resonates with the evidence which indicates that consideration of domestic violence as a private matter is a key barrier in establishing response of HCPs to violence [47, 48].

The outcomes that we assessed are not clinical outcomes, and therefore we cannot ascertain if the size of the differences in these outcomes represent meaningful improvements in the quality of healthcare provided to women experiencing violence. However, the results presented in Tables 7 and 8 indicate changes in practices in terms of identifying women, providing referral and support services, and assessing women’s safety, and represent a substantial shift in practice in the context of the Indian healthcare system, where women experiencing violence usually only receive care for immediate symptoms. In addition, our qualitative findings on HCPs’ perceptions of the impact of their participation in the training on their practices will be reported in a subsequent analysis (in preparation). However, we recognize the complexities of HCP behavior change, and that interventions to change HCP practices are non-linear and complex. HCPs are embedded within health systems, which influence the ability of HCPs to implement skills and practices obtained during training, and specific approaches to behavior change, such as modification of peer group norms and expectations, are more effective than others [49–51]. The finding that attitudes towards the role of HCPs in asking women about violence did not change in our study is relevant in that these attitudes may continue to inform and influence HCP behavior. As such, future refresher trainings and efforts to reinforce HCPs’ quality of practices in response to women experiencing violence should focus on this aspect.

Our study showed greater magnitude of improvement in system level preparedness as compared to individual preparedness. This may indicate that system level changes ensured systems’ support to HCPs but the individual preparedness which is linked to one’s attitude and beliefs showed less improvement. In this study, the intervention not only increased the number of HCPs who inquired about violence but also enhanced the practice of those HCPs who were already doing it before the intervention.

There are different factors responsible for the outcomes observed in our study. In addition to changes at system level, there were certain strategies used for rolling out of training. For example the training implemented by senior HCPs with mentoring, administrative and supervisory roles may have shown to HCPs that their managers were committed to addressing this issue. The interactive approaches such as role plays, games and clinical vignettes are in line with adult learning principles and known to be important in retaining knowledge and skills as shown in a recent scoping review of education intervention programs for HCPs [44]. Further, a mix of doctors, nurses and social workers were trained together which resulted in increased sense of ownership across all cadres and also disrupted professional hierarchies between doctors and nurses. Also, there was increased acceptance of training among HCPs as these were conducted by peers. These training strategies along with the system level changes might have played a role in the positive outcomes of intervention.

The findings of this study should be interpreted in light of certain limitations. Firstly, the study design was pre –post, without a control group. Thus, the changes in outcomes cannot be attributed completely to the intervention. However, given its focus on acceptability and feasibility, we believe that the study design was appropriate at this stage. Secondly, we used self- administered instruments which could have led to social desirability bias in responses, thereby not reflecting true change. Thirdly, our instruments were not previously validated in this context. However, the results presented in this paper are pertaining only to those domains which were found to have medium or high Cronbach’s alpha. Despite these limitations, this study provides robust evidence regarding feasibility and acceptability of a training intervention, combined with health systems-level changes, to support improved HCP knowledge, attitudes and practices for women affected by violence. Important strengths of this study include a large sample size, a low dropout rate and a follow up period, albeit short. The scoping review of training programs for HCPs found mean number of participants in studies of 139.5, and 30% drop-out rate in one- fourth of the included studies [44].

## Conclusions

We found that a training intervention, combined with health-systems level changes, resulted in improvements in knowledge, attitudes and practices of HCPs in tertiary health-care facilities in Maharashtra, India, although changes varied between sub-domains of these constructs. In order to build an effective and sustainable response of healthcare providers to VAW, it is important to introduce system level changes before implementation of the training intervention to create an ecosystem for starting response. The content, design and implementation of the intervention should be evidence-based, implemented within a healthcare setting with potential for systems-level changes, and include content not only on identification of abuse and response but also address attitudes, myths, and misconceptions about violence against women. Repeated in-service trainings are required to bring and sustain changes in HCPs’ attitude and clinical practice. To conclude, training along with system level changes has the potential to strengthen health systems’ response to violence against women.

## Supplementary Information

**Additional file 1.**

## Data Availability

The datasets used and/or analysed during the current study are available from the corresponding author on reasonable request.
