# Supplementary material for: Knowledge, attitudes and practices of health care providers trained in responding to violence against women: a pre- and post-intervention study
Source: BMC Public Health. 2021 Nov 1;21:1973. doi: 10.1186/s12889-021-12042-7 (PMC8561996; doi:10.1186/s12889-021-12042-7)
Supplement: Supplementary file 1 — Additional file 1. [file 12889_2021_12042_MOESM1_ESM.docx]

**Appendix 1: All items and domains in the survey instrument**

| **Construct** | **Domains** | **Items** |
| --- | --- | --- |
| **Knowledge (15 items)** | Clinical knowledge | 1. Repeated unwanted pregnancy? 2. Alcohol or drug abuse? 3. Repeated sexually transmitted infections? 4. Chronic unexplained pain or conditions (e.g. pelvic, headaches)? 5. Frequent injuries? 6. Injuries that do not match the explanation of how they occurred? 7. Depression, anxiety or chronic stress? 8. Thoughts, plans or acts of self-harm or (attempted) suicide 9. Repeated health consultations with no clear diagnosis |
|  | Ways to ask about violence | 1. Are you a victim of domestic violence? 2. Has your partner ever hurt or hit you? 3. Does your partner insult you or threaten you? 4. Many women experience serious problems in their relationships. Have you had any difficulties in your relationship? 5. Are you afraid of your boyfriend/husband? 6. Has anyone else in your family ever hurt you, insulted you or threatened you? |
| **Attitudes (13 items)** | Acceptability of violence | Do you think it is acceptable for a man to hit his wife or partner in the following situations?   1. If she fails to perform her domestic duties. 2. If she disobeys him. 3. If she provokes him or makes him very mad. 4. If she refuses to have sex with him. 5. If she does not look after the children. 6. If he suspects that she is being unfaithful. 7. If he finds out that she is unfaithful. |
|  | Attitude towards asking about violence | 1. Asking patients about domestic violence is an invasion of their privacy 2. It is humiliating to patients to question them about abuse 3. If I ask non-abused patients about domestic violence, they will get very angry 4. I am afraid of offending the patient if I ask about domestic violence 5. Asking about the underlying cause of a patient’s injury does not make a difference to providing quality medical care to the patient 6. The way a couple chooses to resolve a conflict is not my business |
| **Preparedness (15 items)** | Individual preparedness | 1. Identify a woman who is or has been subjected to domestic violence by signs and symptoms she reports 2. Ask a female patient about whether she has experienced domestic violence 3. Provide care to a woman who is or has been subjected to domestic violence 4. Offer validating and supportive statements to a woman subjected to domestic or sexual violence 5. Talk to the woman about her needs and the options she may have 6. Document the history and physical examination findings in patient’s chart 7. Assess the immediate level of danger for a woman after sexual assault and/or domestic violence 8. Help the woman to create a plan to increase her and her children’s safety 9. Refer the woman to support services available within the community (psychological, legal, shelter, etc.) |
|  | System – level preparedness | 1. I have a colleague with whom I can get advice on how to respond to a difficult case of domestic violence if I don’t know what to do 2. I can readily look up information (e.g. either a guide or standard operating procedure on how to manage cases of domestic violence) 3. I have a private space in the facility where I can talk to the woman confidentially about her abuse 4. My supervisor supports me pro-actively asking my patients or clients about whether they are experiencing domestic violence 5. I have names and contact information of people within this facility to whom I can refer the client who discloses violence for additional counselling or psychosocial suppor 6. I have names and contact information of people outside the facility to whom I can refer the woman to for addition psychosocial support |
